# Light stabilizers added to the shell of co-extruded wood/high-density polyethylene composites to improve mechanical and anti-UV ageing properties

**DOI:** 10.1098/rsos.180074

**Published:** 2018-05-09

**Authors:** Chaozheng Liu, Changtong Mei, Bing Xu, Weimin Chen, Cheng Yong, Ke Wang, Qinglin Wu

**Affiliations:** 1 College of Materials and Engineering, Nanjing Forestry University, No.159 Longpan Road, Nanjing 210037, People's Republic of China; 2 School of Renewable Natural Resources, Louisiana State University, Baton Rouge, LA 70803, USA

**Keywords:** wood–plastic composites, co-extrusion, light stabilizers, mechanical property, anti-UV ageing property

## Abstract

Weathering of wood--plastic composites (WPCs) leads to discoloration and cracks, which greatly limits their outdoor application. In this study, light stabilizers (including UV-327, HS-944 and nano-SiO_2_) were added to the shell of a co-extruded high-density polyethylene-based WPC to improve its anti-ultraviolet (UV) ageing properties and simultaneously to maintain its good mechanical properties. The results showed that UV-327 was the most effective light stabilizer for improving the mechanical and anti-UV ageing properties of the composites among the three stabilizers used. WPC samples combined with 2% UV-327 had the highest retention rates in flexural strength and also had the smoothest surface after 2500 h of UV ageing. The samples with 2% UV-327 added had the best protection for discoloration, showing the lowest values of ΔE* (colour difference) and ΔL* (luminescence) in all samples after 2500 h of UV ageing. WPC samples with 2% UV-327 were also oxidized the least after 2500 h of UV ageing. The results reported herein serve to enhance our understanding of the efficiency of light stabilizers in preventing UV degradation of WPCs, with a view to developing co-extruded WPCs with low cost, high anti-UV ageing properties and good mechanical properties for outdoor applications.

## 1. Introduction

In recent years, wood–plastic composites (WPCs), as a replacement for solid wood, have been widely used in outdoor applications such as railings, fencing, decking, and window and door frames [1–3]. However, the durability of WPCs to resist weathering, which results in colour change and loss of mechanical property of the composites after long-term exposure to natural conditions, remains a great concern [4–9]. The weathering of WPCs mainly occurs at the surface of the composites, and an increase in polymer loadings at its surface can help reduce the loss of mechanical properties [10,11]. Lundin [12] weathered photo-stabilized WPCs and monitored the degradation of their mechanical properties. The results demonstrated that the composites lost 33% of their stiffness after 2000 h of ultraviolet (UV) weathering [12]. To improve the anti-UV ageing properties of WPCs, previous work showed that adding light stabilizers to WPCs led to poor interfacial compatibility and poor bonding between the wood fibres and the plastic, resulting in a decrease in the mechanical properties of WPCs [13–15].

Co-extrusion technology has been increasingly applied in the production of WPCs to improve the mechanical and anti-UV ageing properties of the composites [16–19]. Also, co-extrusion technology is considered to be one of the advanced technologies for plastics composite processing [20–24]. For instance, co-extruding a clear hydrophobic high-density polyethylene (HDPE) cap layer over traditional WPCs significantly decreased discoloration during the weathering process. The cap layer absorbed some UV light and reduced the availability of oxygen at the interface of co-extruded WPCs, thereby decreasing the photo-degradation rate [25]. Therefore, one cost-effective method to cope with weathering is to add light-stabilizing treatments to the shell of co-extruded WPCs instead of treating the entire WPCs.

Light stabilizers generally include UV absorbers (UVAs), hindered amine light stabilizers (HALSs) and inorganic UVAs. As UVAs absorb some UV radiation, they significantly reduce the discoloration caused by weathering [26]. Two UVAs, 0.5% UV-326 and UV-531, were used to improve the anti-UV ageing properties of WPCs with a demonstrated protecting effect of the composites from UV degradation [27]. Stark & Matuana [26] investigated the effects of various light stabilizers, including one UVA (UV-328) and two HALSs (Tin770 and Chi944), on the colour change and flexural properties of WPCs. The results indicated that the UVA was a more effective light stabilizer for the composites than the HALSs [26]. Nano-silicon oxide (nano-SiO_2_), an inorganic material, is abundant and cheap. Its preparation process is simple and environmentally friendly. In addition, nano-SiO_2_ has been shown to be a good light reflector in the UV–visible region to inhibit photo-degradation [28]. As a reinforcing agent, a small amount of nano-SiO_2_ (less than 10%) added to the HDPE composite obviously enhanced its tensile strength and impact strength [29]. While that study clearly showed the effectiveness of nano-SiO_2_ in improving the mechanical properties of WPCs, its effect on the anti-UV ageing properties of WPCs was not determined. Thus far, there have been few comparisons of the roles played by individual UVAs, HALSs and nano-SiO_2_ in the UV degradation process for WPCs.

The aim of this study was to find a suitable light stabilizer to improve the mechanical and anti-UV ageing properties of co-extruded WPCs. To achieve this, three kinds of light stabilizers (2% UV-327, HS-944, nano-SiO_2_) were selected and added to the shell of co-extruded WPCs. In particular, the effect of the light stabilizers on the colour, flexural properties, surface morphology and UV degradation of WPCs with a core–shell structure was studied via a series of characterization methods to develop co-extruded WPCs with low cost, high anti-UV ageing properties and good mechanical properties. In addition, the UV ageing and discoloration mechanisms of WPCs are discussed to provide a theoretical basis for the outdoor applications of WPCs.

## 2. Material and methods

### 2.1. Materials

**Figure 1.**
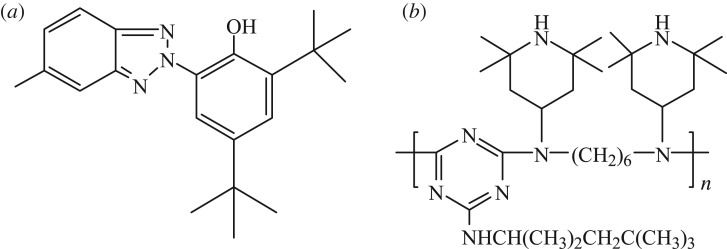
Chemical structure of the UV stabilizers used in this study. (*a*) UV-327 [C_20_H_24_ClN_3_O] and (*b*) HS-944 [(C_35_H_66_N_8_)*_n_*_ = 4–5_].

The HDPE (density, 0.95 g cm^−3^; melting flow index, 0.95 g/10 min; grade, 5000 s) and the coupling agent (maleic anhydride grafted polyethylene; MAH-g-PE) used in this study were supplied by Yangzi Petrochemical Co. Ltd (Nanjing, China). Poplar wood powder with a particle size of 0.134–0.178 mm (60–80 mesh) was purchased from Pucheng Wood Industry Co. (Xuzhou, China). The chemical structures of the UV stabilizers (UV-327 and HS-944) are shown in figure 1. The nano-SiO_2_ (Aerosil® R972) was supplied by Degussa Co. (Hanau, Germany). Its surface was treated with dimethyldichlorosilane and its SiO_2_ content was higher than 99.8%. The lubricants (polyethylene (PE) wax) and talc were purchased from Jupin Chemical Co. Ltd (Dongguan, China).

### 2.2. Preparation of co-extruded high-density polyethylene/wood–plastic composites

The poplar wood powder was dried in an oven at 103 ± 2°C to a moisture content of approximately 1%. The formulation of the core layer composites was: 52% wood powder, 35% HDPE, 9% talc, 2% PE wax and 2% MAH-g-PE, based on the total weight of the composites. The blended core layer composites were placed into a high-speed mixer (type SHR-10A; Huaming Machinery Co., Zhangjiagang, China) for 10 min to disperse the raw materials uniformly and the blends were then transferred to a conical twin screw extruder (type SJZ45/100; Jinwei Machinery Manufacturing Co., Shenzhen, China) for granulation. The formulation of the shell layer composites was: 97.5% HDPE, 2% light stabilizer (UV-327, HS-944 or nano-SiO_2_) and 1.5% PE wax, based on the weight of the HDPE. The blended shell layer composites were mixed in a high mixer for 10 min and then extruded to form granules with a counter-rotating twin screw extruder (type HTY-30; Rubber Machinery Factory Co., Nanjing, China).

Subsequently, the core layer composite blends were fed through a core layer extruder (type SJZ45/100; speed of the main engine, 5 r.p.m.; temperature range, 150–185°C). The blends of the shell layer composites were placed in a shell extruder (type JWS35/25; speed of the main engine, 8 r.p.m.; temperature range, 100–210°C). In addition, WPC was also co-extruded with a clear HDPE shell layer as the control. The cross-sectional dimension was 100 mm × 8 mm (length × width) and the shell thickness was 0.5 mm.

### 2.3. Characterization and testing

According to ASTM G154-06, the samples of WPCs were subjected to UV-accelerated ageing using a fluorescent lamp (type UV-A340). An ageing cycle consisted of UV light irradiation for 8 h at 60 ± 3°C and condensation for 4 h at 50 ± 3°C. The samples were taken out of the test chamber for colour change performance testing after 500 h exposure, and the total duration was 2500 h.

The colour difference of the sample with five replicates after UV ageing was measured by a portable spherical spectrophotometer (type SP60; X-Rite Company, Grand Rapids, MI, USA) based on the CIE 1976 L*a*b* colour system. The wavelength range was 360–700 nm, and five points on each specimen were tested to determine the colour change. The colour change of the specimen was expressed by the following formula [5]:

2.1 $$\Delta E^{*}={\left(\Delta L^{*2}+\Delta a^{*2}+\Delta b^{*2}\right)}^{1/2},$$

where ΔL*, Δa* and Δb* represent the differences between the initial and final values of lightness (L*) and chromaticity coordinates (a* and b*), respectively.

The surface morphology of the sample after 2500 h of UV ageing was observed by scanning electron microscopy (SEM; type Quanta 200; FEI Company, Hillsboro, OR, USA). The surface of the sample was sprayed with gold before SEM observation at an acceleration voltage of 15 kV and the magnification was 300×.

According to GB/T 29418-2012, the flexural properties of the samples after UV ageing were tested by a microcomputer-controlled electronic universal testing machine (type CMT 6104; SANS Material Testing Company, Shenzhen, China). The specimen size was 100 mm × 15 mm × 8 mm (length × width × height). The loading speed was 5 mm min^−1^. The experiment results are presented as the average of the five trials. In this paper, the retention rates of the flexural properties (strength and modulus) were used to judge the mechanical and the anti-UV ageing properties of the specimen. The retention rate of the flexural property denotes the ratio of the flexural strength or the flexural modulus of the composites before and after UV ageing. The formulae for calculating the retention rate of the flexural property are as follows:

2.2 $$\eta (\%)=\frac{S_{2}}{S_{1}}\times 100,$$

where *η* is the retention rate of the flexural strength, *S*_2_ is the flexural strength of the specimen after ageing and *S*_1_ is the flexural strength of the specimen before ageing, and

2.3 $$\lambda (\%)=\frac{E_{2}}{E_{1}}\times 100,$$

where *λ* is the retention rate of the flexural modulus, *E*_2_ is the flexural modulus of the specimen after ageing and *E*_1_ is the flexural modulus of the specimen before ageing.

The functional groups of the samples after UV ageing were analysed by Fourier transform infrared spectroscopy (FTIR; type VERTEX 80V; Bruker Company, Rheinstetten, Germany) with a resolution of 4 cm^−1^, a scanning range of 4000–600 cm^−1^ and a scanning rate of 32 scans min^−1^ [30]. At least five replicate specimens were analysed for each formulation.

The sample was scanned at low resolution from 0 to 1100 eV of binding energy to determine the proportion of the surface element and the change of the oxygen to carbon atomic ratio (O/C). The C1s region was scanned at high resolution from 280 to 300 eV with a 45° incident angle to further analyse the chemical bond in the oxygen and carbon atoms by X-ray photoelectron spectroscopy (XPS; type AXIS UltraDLD; Shimadzu, Osaka, Japan). The C1s peak was diffracted into four subpeaks corresponding to an unoxidized carbon (C1) and various oxidized carbons (C2, C3 and C4). Their relative contents were calculated from the integral area of the corresponding peak. Therefore, the oxidized carbon and unoxidized carbon ratios can be calculated by the following formula [5]:

2.4 $$\frac{C_{\mathrm{ox}}}{C_{\mathrm{unox}}}=\frac{C_{\mathrm{oxidized}}}{C_{\mathrm{unoxidized}}}=\frac{C2+C3+C4}{C1}.$$

## 3. Results and discussion

### 3.1. Mechanical properties

**Figure 2.**
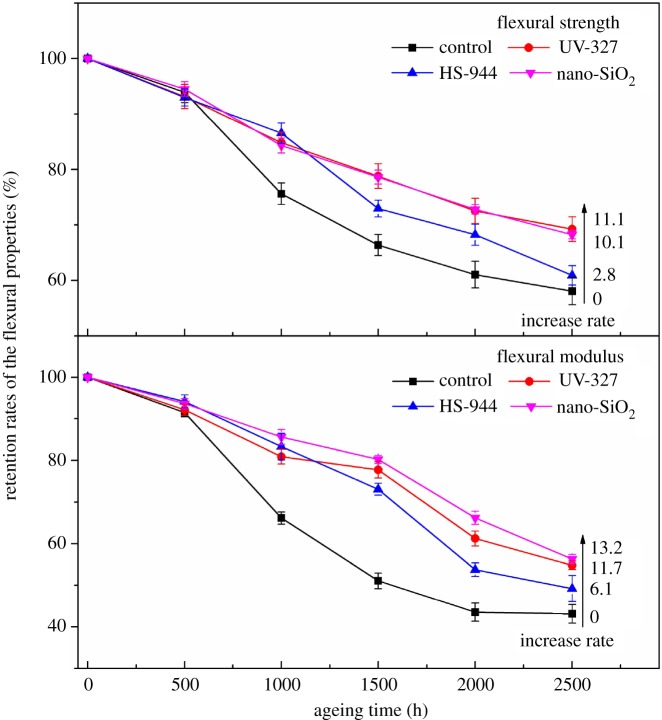
Retention rates of the flexural properties (flexural strength and modulus) of all samples after UV ageing. The retention rate of the flexural property denotes the ratio of the flexural strength or the flexural modulus of the composites before and after UV ageing, obtained by formulae (2.2) and (2.3). The increase rate is the increasing ratio of the retention rate of each sample after 2500 h of UV ageing in comparison with the controls.

The retention rates of the flexural properties of WPCs combined with 2% light stabilizer (UV-327, HS-944 or nano-SiO_2_) after UV weathering are shown in figure 2. The retention rates of the flexural properties of all samples showed a tendency to decrease with test duration, and those of the samples with added light stabilizers were higher than the controls. These results indicated that the accelerated UV weathering resulted in loss of the flexural properties of the composites and light stabilizers effectively protected the composites from UV ageing. During 1000–2500 h of UV weathering, the retention rate of the flexural property of WPCs with 2% HS-944 decreased sharply, which was mainly due to the rapid decrease of HS-944 in the shell. And this is because alkaline groups in the HS-944 easily reacted with the acidic groups in the lignin, which was exposed due to the cracked surface in the later stages of UV ageing, thus reducing the light stability of the composites [31]. The retention rate of the flexural strength of the samples with 2% UV-327 and the retention rate of the flexural modulus of the samples with 2% nano-SiO_2_ were both higher than those of the other formulations after 2500 h of UV ageing. These results indicated that adding HS-944 to the shell provided good protection for the flexural properties of the composites, especially in the early period of UV ageing, and the UV-327 and nano-SiO_2_ both protected the flexural properties well in the later stages of UV ageing. In addition, after 2500 h of UV exposure, the retention rates of the flexural strength of the samples with 2% UV-327, HS-944 or nano-SiO_2_ were, respectively, increased by 11.1%, 2.8% and 10.1% in comparison with that of the controls, and the retention rates of the flexural modulus were increased by 11.7%, 6.1% and 13.2%, respectively. Therefore, UV-327 and nano-SiO_2_ were more effective light stabilizers in protecting the flexural strength and modulus of the composites, especially when the duration exceeded 1000 h. These results also implied that adding light stabilizers to the shell of WPCs prevented the composites from absorbing UV light to produce degradation, which protected the flexural properties of the composites.

### 3.2. Anti-ultraviolet ageing properties

#### 3.2.1. Colour measurements

**Figure 3.**
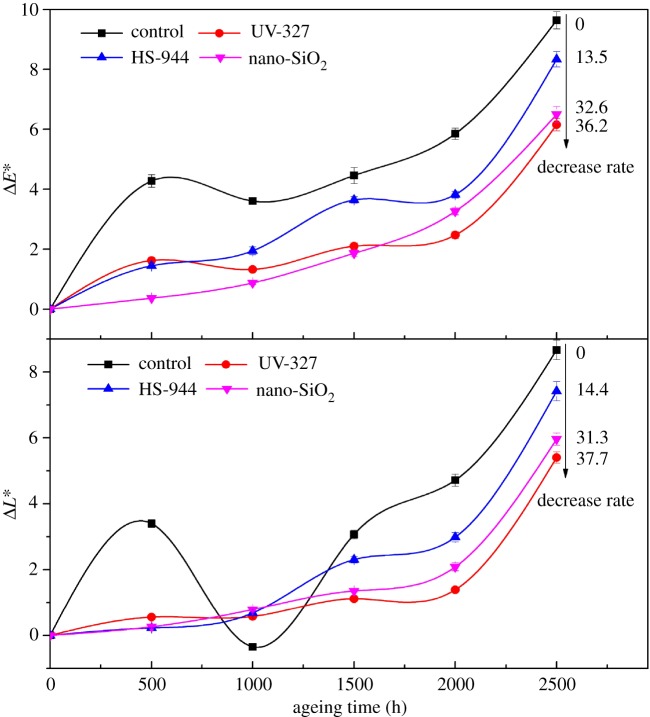
Colour changes of WPCs with 2% UV-327/HS-944/nano-SiO_2_ after UV-accelerated ageing. Values of the ΔE* were obtained by using formula (2.1). The decrease rate is the decreasing ratio of the ΔE* (colour difference) and the ΔL* (luminescence) of each sample after 2500 h of UV ageing in comparison with the controls.

Figure 3 shows the colour change of WPCs with 2% light stabilizer (UV-327, HS-944 or nano-SiO_2_) after UV-accelerated ageing. The decrease rate is the decreasing ratio of the ΔE* (colour difference) and the ΔL* (luminescence) of each sample after 2500 h of UV ageing in comparison with those of the controls. The ΔE* and ΔL* values of all samples increased as the duration increased, indicating that the surface of the WPCs faded. These results were attributed to the composite surfaces undergoing photo-degradation, resulting in a decrease in the density of the entanglement in the non-crystalline region [32]. Thus, the surface brightness of the WPCs increased. The samples with 2% UV-327 had the smallest changes in ΔE* and ΔL* values after 2500 h of UV ageing. This result indicated that the UV-327 acted as an anti-ageing agent to effectively absorb UV light and improve the fade phenomenon of the composites. However, the increase in the ΔE* and ΔL* values of the samples with added light stabilizers also showed that the light stabilizers did not completely remove the free radicals formed in the photo-degradation process to prevent the formation of chromophore functional groups [10]. The ΔE* values of the samples with 2% UV-327, HS-944 or nano-SiO_2_ after 2500 h of UV ageing were lower than that of the control by 36.2%, 13.5% and 32.6%, respectively. Also, the ΔL* value decreased by 37.7%, 14.4% and 31.3%. Thus, the addition of UV-327 had a better protective effect on the degradation of HDPE molecular chains and the discoloration of the composites surface than HS-944 or nano-SiO_2_. In addition, the ΔE* and ΔL* values of the control during the duration of 500–1000 h decreased significantly, especially when the ΔL* value reduced to a negative value. This was due to the slow degradation of the HDPE in the shell layer and the *p*-benzoquinone chromogenic groups of the lignin in the core layer, which resulted in the colours of the composites being temporarily tilted to dark [25].

#### 3.2.2. Surface morphology

**Figure 4.**
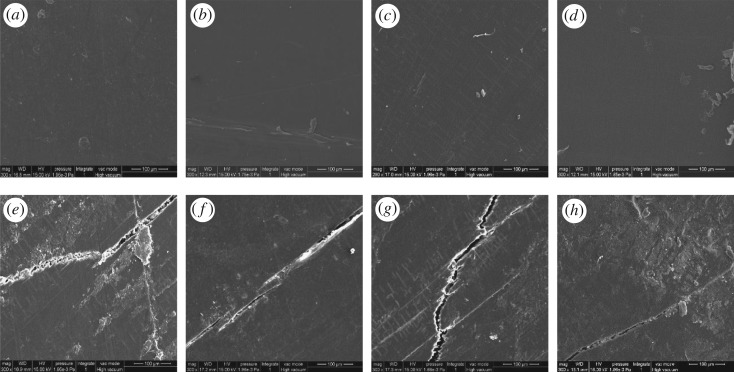
Surface morphologies of WPCs with different light stabilizers after 2500 h of UV ageing. Samples (*a*,*e*): control; (*b*,*f*): 2% UV-327; (*c*,*g*): 2% HS-944; (*d*,*h*): 2% nano-SiO_2_ after 0 h and 2500 h of UV exposure, respectively.

Figure 4 shows the surface morphology of WPCs with 2% light stabilizer (UV-327, HS-944 or nano-SiO_2_) after UV-accelerated ageing. All samples demonstrated a smooth surface before UV ageing. The core layer was wrapped with the HDPE in co-extruded WPCs. With increasing test duration, the surface of the controls gradually showed an increased number of cracks. These cracks in the control surfaces became more extensive than the other samples after UV exposure for 2500 h. In addition, although all of the cracks enlarged obviously after 2500 h of UV ageing, the surface cracks of the samples with added light stabilizers were less obvious than those in the controls. The surface of the samples with 2% UV-327 was the smoothest in all samples. These results indicated that the light stabilizers protected WPCs against damage by UV radiation and the samples with UV-327 had the best effects of surface protection compared with the samples with HS-944 or nano-SiO_2_. However, as the duration increased, the effect of this surface protection decreased and cracks eventually appeared on the surface of the composites. Thus, light stabilizers could absorb UV light to transform it into thermal energy, which delayed the degradation of the composites [15]. Nevertheless, once the plastic shell cracked, the wood fibre in the core layer was exposed to optothermal and water complex ageing. Simultaneously, the basic groups of light stabilizers reacted with the acidic groups of lignin to reduce the light stability of the composites, thereby leading to the degradation of the composites [32]. Therefore, the light stabilizers at the surface of the samples gradually disappeared as the test duration increased, which led to the loss of prevention of degradation of the composites and the presence of cracks at the surface of the composites.

#### 3.2.3. Surface chemical properties

**Figure 5.**
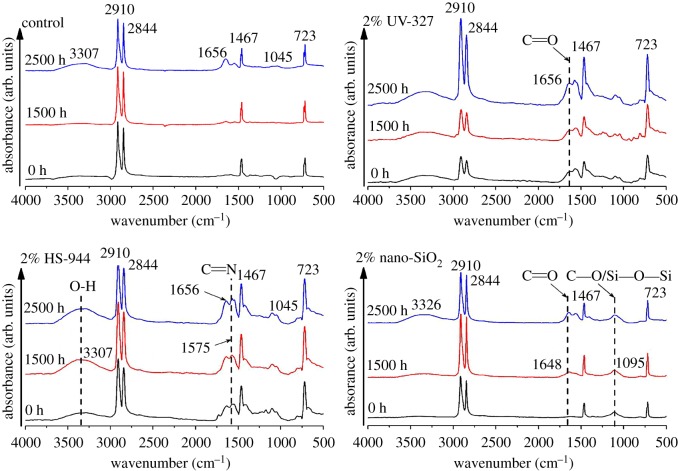
FTIR spectra of WPCs with light stabilizers (2% UV-327/HS-944/nano-SiO_2_) after 0 h, 1500 h and 2500 h of UV weathering. Their wavenumbers along with the assignments of the corresponding functional groups are marked.

Figure 5 shows the FTIR spectra of WPCs with 2% light stabilizer (UV-327, HS-944 or nano-SiO_2_) after UV ageing. Distinct absorption peaks of the samples were observed at around 3307, 2910, 2844, 1656, 1575, 1467, 1045 and 723 cm^−1^. Table 1 lists their wavenumbers along with the assignments of the corresponding functional groups and vibrational types [33–37]. It can be seen that the intensity of the peak at 3307 cm^−1^ (–OH) was obviously enhanced after 2500 h of UV ageing, which was mainly due to the hydroxyl in the HDPE and the hydroperoxide (ROOH) in the wood fibres of the exposed core layer [27]. The intensity of the peak at 1656 cm^−1^ had also been enhanced after 2500 h of UV ageing. This result was attributed to the C=O vibrational peak formed by the carbonyl (C=O) in the lignin and the HDPE fracture chain [3]. These results indicated that the HDPE in the shell after 2500 h of UV ageing had produced a fracture and the lignin in the core layer was also exposed due to the occurrence of photo-oxygen degradation [25]. The intensity of the peak around 1575 cm^−1^ corresponded to the characteristic absorption of the conjugated imino group C=N in light stabilizers, and these features decreased gradually after 2500 h of UV exposure. The results indicated that light stabilizers at the surface of the WPCs were consumed and that they captured or cleared the peroxidative free radicals in the UV ageing process, which delayed the oxidative degradation of the WPC surface [10]. The intensity of the peak at 1045 cm^−1^ that corresponded to the C–O stretching in cellulose and hemicellulose was also enhanced after UV ageing. This result indicated that the presence of light stabilizers prevented the loss of cellulose and hemicellulose at the composite surface [38]. It was found from FTIR of the samples with 2% nano-SiO_2_ that the bond of Si–O–Si with a peak at 1095 cm^−1^ was introduced into the shell to enhance the surface activity, thereby reducing the light stability and protecting the surface of the composites from photo-degradation [39]. This was consistent with the surface of the samples with 2% nano-SiO_2_ being smoother than the controls in SEM analysis.

**Table 1.** Wavenumbers of peaks used for FTIR analysis and the corresponding functional groups and vibrational types.

wavenumber (cm^−1^)	functional group	vibrational type
3307	─OH	─OH stretching
2910 or 2844	─CH_2_−	C─H stretching
1656	C=O(conjugated)	C=O stretching conjugated with benzene
1575	C=N(conjugated)	C=N stretching conjugated with benzene
1467	─CH_2_−	CH_2_ bending, crystalline or amorphous
1045	C─O	C─O stretching
723	─CH_2_−	CH_2_ rocking, crystalline or amorphous

XPS was used to study the surface chemical properties of all samples, including the compositions of the atomic elements (C, O, N and Si) and chemical groups. Table 2 shows the relative contents of the surface elements in WPCs after UV weathering. The O/C ratio was used to indicate the degree of oxidation at the sample surface. It can be seen that the surface carbon element contents of all samples were decreased after 2500 h of UV ageing, while the surface oxygen element contents were increased. And thus the O/C ratio increased for all of the samples after 2500 h of UV exposure. These results indicated that all sample surfaces were oxidized after UV weathering. In addition, the O/C ratios of the samples with added light stabilizers were all lower than those of the controls before and after UV ageing. This result implied that adding light stabilizers to the shell effectively hindered the photo-oxidation of the sample surface.

**Table 2.** Relative contents of the surface elements of WPCs after UV ageing. Ratios of the oxidized carbon and the unoxidized carbon (C_ox/unox_), obtained by using formula (2.4) with the experimental data.

	unweathered samples				samples weathered for 2500 h			
elements	control	UV-327	HS-944	nano-SiO_2_	control	UV-327	HS-944	nano-SiO_2_
C	79.3	85.1	87.2	83.8	67.2	72.9	74.4	72.9
O	19.5	10.5	7.9	10.2	30.4	19.9	18.1	20.2
N	0.5	0.9	1.3	1.2	1.3	5.3	3.6	5.2
Si	0.7	3.5	3.6	4.8	1.1	1.9	3.9	1.7
O/C	0.245	0.123	0.090	0.122	0.452	0.273	0.243	0.277
C_ox/unox_	0.285	0.241	0.192	0.230	0.515	0.389	0.418	0.395

**Figure 6.**
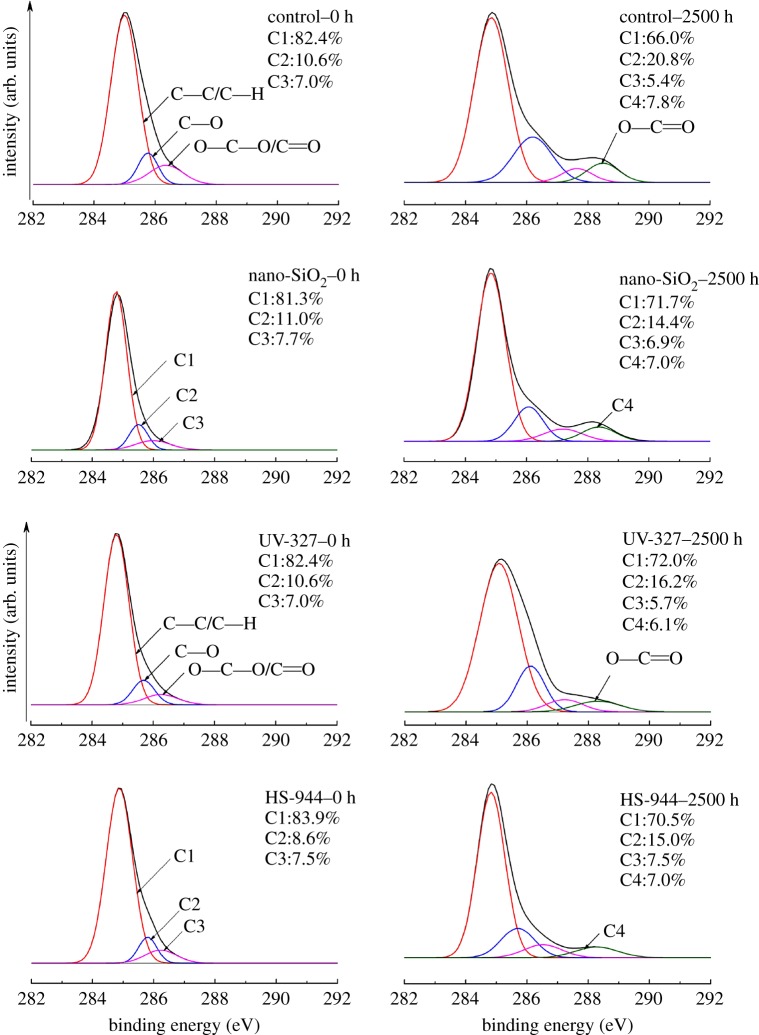
XPS spectra of the C elements on the surface of WPCs with 2% UV-327/HS-944/nano-SiO_2_ after UV ageing. The C1s peak was diffracted into four subpeaks corresponding to an unoxidized carbon (C1, –C–C– or –C–H at 284.6 eV) and various oxidized carbons (C2, –C–O at 285.9 eV; C3, –C=O at 287.7 eV; C4, O–C=O at 288.8 eV). Their relative contents were calculated from the integral area of the corresponding peak.

Figure 6 shows the types and numbers of carbon–oxygen bonds at the surface of all samples after UV ageing. The C1s spectra, which were further deconvoluted into four carbon-related chemical groups (–C–C– or –C–H at 284.6 eV; –C–O at 285.9 eV; –C=O at 287.7 eV; O–C = O at 288.8 eV), are presented. The C1 peak did not possess a carbon–oxygen bond, but C2, C3 and C4 all did. As shown in table 2, weathering obviously increased the C_ox/unox_ ratio for all samples, demonstrating that substantial surface oxidation occurred after 2500 h of UV ageing [6]. Also, the peak intensity corresponding to C1 significantly decreased while the intensity of the C2 peak increased, and the C4 peak appeared after 2500 h of UV exposure. The oxidation of C1 (C–C/C–H) mainly produced C–O chemical bonds of C2. C1 and part C3 were further oxidized to produce C4 chemical bonds [40]. These results indicated that the oxidation reaction occurred after 2500 h of UV ageing, leading to O–C=O chemical bonds. The C_ox/unox_ ratio of the samples with 2% UV-327, HS-944 or nano-SiO_2_ was increased by 14.8%, 22.6% and 16.5%, respectively, after 2500 h of UV ageing and the C_ox/unox_ value of the controls was increased by 23%. These results showed that adding UV-327 to the shell significantly reduced the photo-oxidative degradation of the composites and thus improved the anti-UV ageing properties of the core–shell structural WPCs.

## 4. Conclusion

The anti-UV ageing properties of co-extruded WPCs were improved by adding light stabilizer (UV-327, HS-944 or nano-SiO_2_) to its shell, and simultaneously its mechanical properties were also maintained. The UV-327 and nano-SiO_2_ demonstrated the best effect on protecting the flexural strength and modulus of the composites compared with the other additives used, showing an increase in the retention rates of flexural strength and modulus of 11.1% and 13.2%, respectively, in comparison with those of the controls after 2500 h of UV ageing. Also, the UV-327 provided a better protection against the surface discoloration of the composites, demonstrating the lowest values of the luminescence (ΔL*) and colour difference (ΔE*) in all samples after 2500 h of UV ageing. This was consistent with the SEM images that WPC with 2% UV-327 had the smoothest surface after 2500 h of UV ageing. In addition, the UV-327 was a more effective light stabilizer than the others in improving the photo-oxidative degradation of the composites, showing the lowest increase in the C_ox/unox_ ratio of the samples with 2% UV-327 by 14.8% in all samples after 2500 h of UV ageing. The obtained results provide a theoretical basis for developing co-extruded WPCs with low cost, high anti-UV ageing properties and good mechanical properties by adding light stabilizers into the shell for outdoor applications.

## Data accessibility

Our data are deposited at Dryad (https://doi.org/10.5061/dryad.m810q41) [41].
